# Cocoa Pulp Juice as a Fermentation Substrate for Potential Probiotic *Lacticaseibacillus paracasei* WU2502: Genomic Characterization, Metabolite Profiling, and Antimicrobial Activity Against Foodborne Pathogens

**DOI:** 10.3390/ijms27219795

**Published:** 2026-10-31

**Authors:** Watcharapong Mitsuwan, Phoomjai Sornsenee, Morteza Saki, Chonticha Romyasamit

**Affiliations:** 1 Akkhraratchakumari Veterinary College, Walailak University, Nakhon Si Thammarat 80160, Thailand; watcharapong.mi@wu.ac.th; 2 Research Center in One Health, Walailak University, Nakhon Si Thammarat 80160, Thailand; 3 Research Center of Excellence in Innovation of Essential Oil Center of Excellence in Innovation of Essential Oil and Bio-Active Compound, Walailak University, Nakhon Si Thammarat 80160, Thailand; 4 Department of Family and Preventive Medicine, Faculty of Medicine, Prince of Songkla University, Hat Yai, Songkhla 90110, Thailand; poomjai@s.psu.ac.th; 5 Department of Microbiology, Faculty of Medicine, Ahvaz Jundishapur University of Medical Sciences, Ahvaz, Iran; mortezasaki1981@gmail.com; 6 Department of Medical Technology, School of Allied Health Sciences, Walailak University, Nakhon Si Thammarat 80160, Thailand; 7 Research Center for Microbiome, Systems Biology, and Medical Innovation, Walailak University, Nakhon Si Thammarat 80160, Thailand; 8 Research Center in Tropical Pathobiology, Walailak University, Nakhon Si Thammarat 80160, Thailand

**Keywords:** *Lacticaseibacillus paracasei*, cocoa pulp juice, potential probiotic, genome mining, antimicrobial activity, by-product valorization

## Abstract

Cocoa pulp juice is an underutilized by-product generated during cocoa bean fermentation that is rich in fermentable sugars and phytochemicals but remains largely unexplored as a substrate for microbial fermentation. This study aimed to evaluate cocoa pulp juice as a sustainable fermentation substrate for the potential probiotic *Lacticaseibacillus paracasei* WU2502 using whole-genome sequencing (WGS), carbohydrate-active enzyme (CAZyme) analysis, untargeted LC–QTOF-MS-based metabolite profiling, and antimicrobial evaluation. *L. paracasei* WU2502 showed substantial growth in cocoa pulp juice, reaching a maximum viable count of 10.70 log CFU/mL in 25% juice and >11 log CFU/mL in 50% and 100% juice, while the pH decreased from approximately 3.9–4.0 at Day 0 to 3.1–3.2 during fermentation. Fermented cocoa pulp juice showed antibacterial activity against *Bacillus cereus*, *Staphylococcus aureus*, *Salmonella enterica* serovar Typhimurium and *Escherichia coli* O157:H7 with MIC and MBC values of 12.5–25% (*v*/*v*). In a preliminary qualitative assessment, neutralization reduced the observed inhibitory activity, whereas Proteinase K treatment produced little apparent change, suggesting a possible pH-dependent contribution to the antimicrobial activity. WGS resulted in a 3.02 Mb draft genome with a GC content of 46.27%, 3114 coding sequences and genes relevant to glucose metabolism, acid tolerance, oxidative stress response and antimicrobial activity. Genome mining identified putative bacteriocin-associated regions containing Carnocin CP52- and Thermophilin A-related sequences and a RiPP-like biosynthetic region. No acquired antimicrobial resistance genes or virulence-associated factors were detected. CAZyme analysis revealed glycoside hydrolases and glycosyltransferases that may be involved in carbohydrate metabolic pathways and adaptation to plant-derived substrates. LC–MS/MS analysis of unfermented cocoa pulp juice provided baseline chemical characterization of the starting substrate, with representative putative annotations including sucrose, maltotriose, myo-inositol, citric acid, pyroglutamic acid, and choline. These findings support the feasibility of cocoa pulp juice as a fermentation substrate for *L. paracasei* WU2502 and provide a basis for further evaluation of this strain and fermented matrix for potential food applications.

## 1. Introduction

The global cocoa (*Theobroma cacao* L.) processing sector produces substantial agricultural by-products, presenting considerable economic and environmental challenges. Although cocoa beans are the primary raw material for chocolate production, the surrounding mucilaginous pulp, which accounts for approximately 10% of the fresh fruit weight, is often underutilized during cocoa processing [1]. During cocoa fermentation, the mucilaginous pulp surrounding cocoa beans liquefies and drains as an exudate known as cocoa pulp juice, cocoa honey, or “sweatings”. Large quantities of this by-product are generated during cocoa processing, and its improper disposal can contribute to environmental pollution due to its high chemical oxygen demand (COD) and biochemical oxygen demand (BOD) [2,3]. Nevertheless, cocoa pulp juice represents a valuable yet underutilized substrate for functional fermentation. It is naturally rich in fermentable sugars, primarily glucose, fructose, and sucrose, and contains organic acids, mainly citric acid, resulting in an acidic pH ranging from 2.7 to 4.0 [4,5]. In addition, cocoa pulp juice contains various phytochemicals, minerals, volatile compounds, and polyphenols that may support microbial growth and contribute to biological activities [6]. These characteristics suggest that cocoa pulp juice could serve as a promising natural substrate for lactic acid bacterial fermentation without requiring synthetic nutrient supplementation.

The valorization of food industry by-products through fermentation with lactic acid bacteria (LAB) has gained increasing attention, particularly through the application of potential probiotic strains. Among these, *L. paracasei* (formerly *Lactobacillus paracasei*) [7] has been widely investigated as a source of potential probiotic strains for food, dairy, and pharmaceutical applications because certain strains have demonstrated health-promoting properties, including gastrointestinal homeostasis, immunomodulatory effects, and the production of bioactive metabolites [8,9]. In recent years, research interest has increasingly shifted toward indigenous or “local isolate” strains obtained from diverse ecological niches, as some of these strains have demonstrated promising technological and functional properties, such as enhanced tolerance to physiological stress, acidic conditions, and phenolic-rich environments, making them attractive candidates for food fermentation and functional food development [10,11].

In our previous study, *L. paracasei* WU2502 was isolated from the natural fermentation of Asian palmyra palm (*Borassus flabellifer* L.) sap used for traditional palm sugar (“Namtan Tanod”) production in Thailand [10]. The results demonstrated that this strain possesses promising probiotic characteristics, including strong cell surface hydrophobicity, high auto-aggregation ability, survival under simulated gastrointestinal conditions, and a non-hemolytic safety profile [10]. These phenotypic characteristics support its potential as a probiotic candidate [10]. However, limited information is currently available regarding the genomic background and functional genetic traits of this strain. Comprehensive genomic characterization may provide valuable insights into genes associated with carbohydrate metabolism, stress adaptation, antimicrobial compound production, and other probiotic-related functions. In particular, whole-genome sequencing (WGS) can improve the understanding of how *L. paracasei* WU2502 adapts to and utilizes complex plant-derived substrates.

The functional importance of fermented food matrices lies in their potential to provide natural antimicrobial activity against foodborne pathogens and to support food preservation strategies. According to the World Health Organization (WHO), approximately 600 million people suffer from foodborne diseases annually, resulting in nearly 420,000 deaths worldwide, with 30% occurring among children [12]. Pathogenic bacteria, including *S. aureus*, *E. coli*, and *Salmonella* spp., are among the leading causes of foodborne infections and outbreaks, contributing substantially to public health burdens and economic losses [13,14]. Furthermore, the rapid emergence and dissemination of multidrug-resistant (MDR) foodborne pathogens, largely driven by the excessive use of antibiotics in agriculture and veterinary medicine, have significantly reduced the effectiveness of conventional food preservation strategies [14]. Moreover, growing consumer demand for “clean-label” foods has increased interest in reducing the use of synthetic preservatives, such as benzoates and sorbates, and in exploring natural alternatives for food preservation [15]. Consequently, increasing attention has been directed toward the development of natural and sustainable antimicrobial alternatives. Probiotic-fermented agricultural matrices are particularly promising because they can suppress foodborne pathogens through the combined effects of organic acids, hydrogen peroxide, and bacteriocin-like inhibitory substances, while simultaneously enhancing the food matrix with beneficial bioactive metabolites [16].

The microbial ecology of cocoa fermentation has been well studied; however, the relationship between strain-level genomic traits of LAB and their performance in cocoa pulp juice remains poorly understood [17]. This is particularly relevant for *L. paracasei*, which exhibits substantial diversity in carbohydrate-utilization genes among strains [18]. Therefore, this study aimed to: (1) evaluate growth and acidification during cocoa pulp juice fermentation; (2) characterize genomic traits related to carbohydrate utilization, stress adaptation, and antimicrobial biosynthetic potential; (3) evaluate antimicrobial activity against selected foodborne pathogens; and (4) characterize representative metabolites in unfermented cocoa pulp juice using untargeted LC–QTOF-MS.

## 2. Results

### 2.1. Viability of L. paracasei WU2502 in the Fermentation Medium Containing Cocoa Pulp Juice

The viability of *L. paracasei* WU2502 in different cocoa pulp juice formulations during fermentation is presented in Table 1. Three-way mixed ANOVA showed a significant juice concentration × glucose × incubation time interaction (F(6,36) = 123.61, *p* < 0.001). The bacterial population increased markedly during the fermentation period in all tested formulations. Initial bacterial counts ranged from 5.10 ± 0.09 to 5.53 ± 0.03 log CFU/mL on day 0. After incubation, the viable counts increased progressively and reached the highest levels on days 4 and 6. Among the tested formulations, 100% cocoa pulp juice supported the highest bacterial growth, with viable counts reaching 11.46 ± 0.00 log CFU/mL on day 6. Glucose supplementation influenced viable counts in a formulation- and time-dependent manner but did not consistently increase bacterial growth across all juice concentrations. However, bacterial viability slightly decreased in some formulations after prolonged incubation.

The pH values of fermented cocoa pulp juice formulations are shown in Table 2. The initial pH values ranged from 3.88 ± 0.02 to 4.00 ± 0.15 on day 0 and gradually decreased throughout the fermentation process. The lowest pH values were observed on day 4, ranging from 3.08 ± 0.01 to 3.21 ± 0.09, indicating progressive acidification during fermentation. Slight increases in pH were observed on day 6 in some formulations.

### 2.2. Antimicrobial Activity of Fermented Cocoa Pulp Juice Using L. paracasei WU2502 Against Pathogens

The antimicrobial activity of fermented cocoa pulp juice against pathogenic bacteria was preliminarily evaluated using the agar well diffusion assay, as presented in Table 3. All fermented cocoa pulp juice formulations exhibited inhibitory activity against the tested pathogens, including *B. cereus*, *S. aureus*, *S*. Typhimurium, and *E. coli* O157:H7. The inhibition zones ranged from 10.00 to 14.67 mm, with the smallest inhibition zone (10.00 mm) observed against *S. aureus* in the glucose-supplemented treatment. The strongest antibacterial activity was observed against *B. cereus* and *S*. Typhimurium, with inhibition zones of 14.67 mm in the 100% cocoa pulp juice formulations. In contrast, *S. aureus* showed comparatively lower susceptibility, with inhibition zones ranging from 10.00 to 12.33 mm.

The MIC and MBC values of fermented cocoa pulp juice against the tested pathogens are summarized in Table 3. The fermented cocoa pulp juice demonstrated antibacterial activity against all tested pathogens, with MIC values of 12.5% (*v*/*v*) for all bacterial strains. The MBC values against *B. cereus* and *S*. Typhimurium were 12.5% (*v*/*v*), whereas higher MBC values of 25% (*v*/*v*) were observed against *S. aureus* and *E. coli* O157:H7. These findings indicate that the CFS exhibited measurable bactericidal activity against the tested pathogens, with MBC values of 12.5% (*v*/*v*) against *B. cereus* and *S*. Typhimurium and 25% (*v*/*v*) against *S. aureus* and *E. coli* O157:H7.

In a preliminary qualitative assessment, neutralization of the CFS markedly reduced the observed inhibitory activity, whereas Proteinase K treatment produced little apparent change. These results suggest a possible pH-dependent contribution to the antimicrobial activity. However, because these preliminary assays were not designed for quantitative mechanistic evaluation, the relative contributions of acidic conditions, proteinaceous compounds, and other antimicrobial factors could not be determined.

### 2.3. Whole-Genome Characterization of Lacticaseibacillus paracasei WU2502

**Figure 1:**
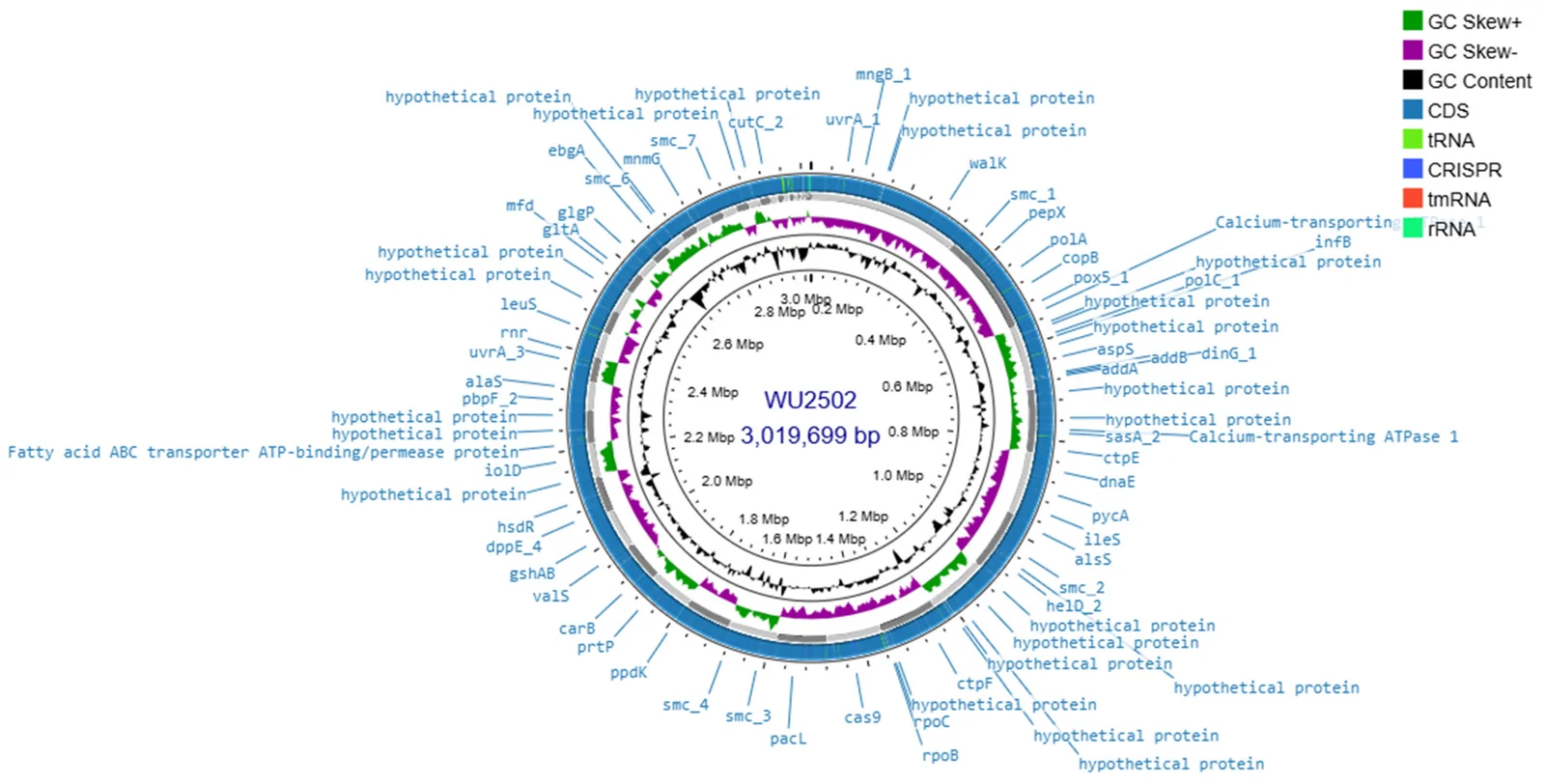
Circular genome maps of *L. paracasei* WU2502.

WGS of *L. paracasei* WU2502 generated a draft genome of 3,019,699 bp with a GC content of 46.27% (Figure 1 and Table 4). The assembled genome comprised 50 contigs, with an N50 value of 108,079 bp and an L50 value of 10. Genome-quality evaluation indicated 100% completeness and 0% contamination. No plasmid sequences were detected in the assembled genome. Analysis using mobileOG-db detected 139 mobile genetic element-associated hits, comprising 10 integration/excision, 44 replication/recombination/repair, 62 phage, 8 stability/transfer/defense, and 15 transfer-associated hits. Genome annotation using the RASTtk pipeline identified 3114 protein-coding sequences (CDSs), 50 transfer RNA (tRNA) genes, and 5 ribosomal RNA (rRNA) genes. Among the predicted proteins, 2086 were assigned putative biological functions, whereas 1028 were annotated as hypothetical proteins. Functional annotation further identified 662 proteins with Enzyme Commission (EC) assignments, 542 proteins associated with Gene Ontology (GO) terms, and 449 proteins linked to KEGG metabolic pathways, suggesting considerable metabolic versatility of strain WU2502.

Average nucleotide identity (ANI) analysis showed 98.24% identity between strain WU2502 and the reference genome *L. paracasei* subsp. *paracasei* GCF_000829035.1 (ASM82903v1). This value exceeds the commonly accepted 95–96% ANI threshold for prokaryotic species delineation, supporting the assignment of WU2502 to *L. paracasei* (Table 4). The circular genome map demonstrated a balanced distribution of coding sequences on both forward and reverse strands, together with RNA genes, GC content, and GC skew across the genome (Figure 1). Putative prophage regions were detected in the draft genome of *L. paracasei* WU2502; however, these regions were not further characterized in the present study. One putative CRISPR locus was detected in the draft genome of *L. paracasei* WU2502; however, the CRISPR-Cas system was not further characterized in the present study. In addition, functional annotation revealed genes potentially associated with carbohydrate metabolism, environmental stress adaptation, and antimicrobial-related functions, supporting the probiotic and fermentative potential of strain WU2502 in plant-derived substrates.

No acquired antimicrobial resistance genes (ARGs) or major virulence-associated determinants were detected by genome screening using the applied bioinformatic tools. However, these findings should not be interpreted as a comprehensive genomic safety assessment of strain WU2502. Furthermore, one putative CRISPR locus was identified in the draft genome, although the CRISPR-Cas system was not further characterized in the present study (Table 4 and Figure 1).

**Figure 2:**
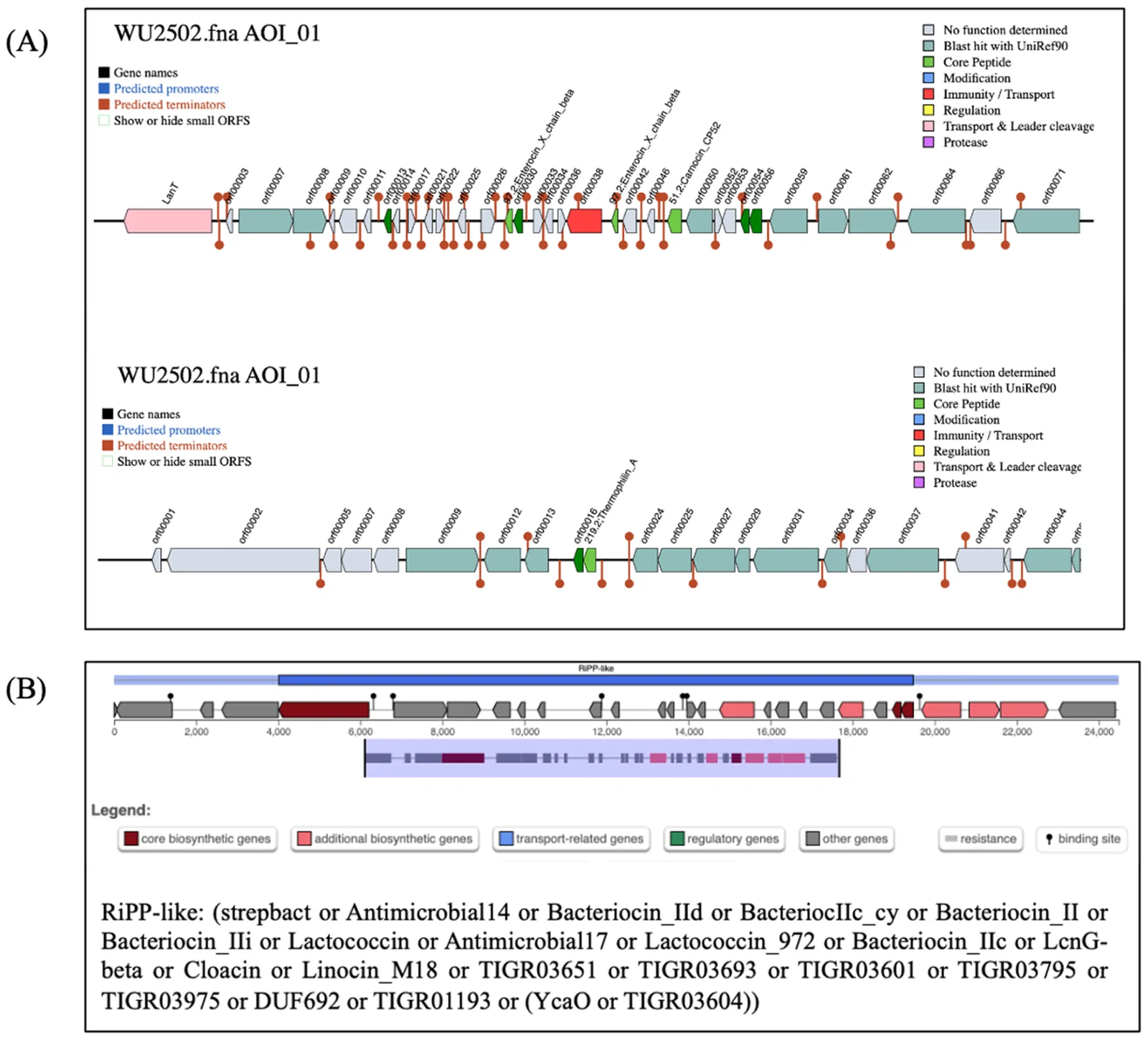
Genomic organization of putative bacteriocin-associated and RiPP-like biosynthetic gene clusters identified in *Lacticaseibacillus paracasei* WU2502. (**A**) BAGEL4-predicted regions containing putative Carnocin CP52-related (**upper panel**) and Thermophilin A-related (**lower panel**) sequences. The “AOI_01” labels shown in both panels are automatically generated BAGEL4 area-of-interest designations for the respective outputs. (**B**) antiSMASH-predicted RiPP-like biosynthetic gene cluster identified in *L. paracasei* WU2502.

To further characterize its predicted antimicrobial potential, genome mining analysis was performed using BAGEL4 and antiSMASH platforms. BAGEL4 analysis identified putative bacteriocin-associated biosynthetic genes encoding Carnocin CP52 and Thermophilin A within the *L. paracasei* WU2502 genome (Figure 2A). In parallel, antiSMASH analysis revealed a ribosomally synthesized and post-translationally modified peptide (RiPP)-like biosynthetic gene cluster containing multiple biosynthetic, transport-related, and regulatory genes potentially involved in antimicrobial peptide production (Figure 2B). Comparative analysis demonstrated similarity to previously reported antimicrobial peptides, including gassericin T, gassericin E, coagulin, carnobacteriocin XY, salivaricin CRL1328, and lactococcin G. These genomic findings indicate the predicted antimicrobial biosynthetic potential of WU2502; however, the contribution of these predicted regions to the observed antimicrobial activity was not directly demonstrated.

Functional genome annotation also identified several putative probiotic-associated genes involved in acid tolerance, environmental stress response, oxidative stress response, osmotic stress adaptation, carbohydrate utilization, and antimicrobial activity (Table 5). Notably, the genome contained genes encoding F0F1 ATP synthase subunits (*atpA–G*), molecular chaperones (*dnaK*, *dnaJ*, *clpB*, and *clpP*), oxidative stress-related proteins (*sodA*, *tpx*, and *npr*), and carbohydrate transport/metabolism systems. In addition, genes associated with adhesion and exopolysaccharide biosynthesis were identified, suggesting the potential adaptation of strain WU2502 to gastrointestinal and plant-derived fermentation environments.

### 2.4. CAZyme Analysis

CAZyme analysis revealed that *L. paracasei* WU2502 possesses a diverse repertoire of carbohydrate-active enzymes, including GHs, GTs, CEs, and PLs. The predominant CAZyme families included GT2, GT4, GH25, GH13_31, and GH1. GT2 and GT4 glycosyltransferases are potentially involved in glycan and exopolysaccharide biosynthesis, whereas GH25, GH13_31, and GH1 represent glycoside hydrolase families associated with carbohydrate or glycan processing (Table S1). The presence of GH32 family enzymes further indicates predicted genetic potential related to sucrose- and fructose-associated carbohydrate metabolism. These genomic features should be interpreted as predicted functional potential rather than direct evidence of substrate utilization during cocoa pulp juice fermentation.

### 2.5. Baseline LC–QTOF-MS Characterization of Unfermented Cocoa Pulp Juice

Untargeted LC–QTOF-MS analysis was performed to provide baseline chemical characterization of the unfermented cocoa pulp juice used as the starting fermentation substrate. The retained MassHunter compound-identification reports contained 35 compound-identification hits in positive-ionization mode and 23 hits in negative-ionization mode (Table S2). These numbers represent compound-identification hits generated by the applied analytical workflow rather than the total number of LC–MS features detected prior to annotation.

Representative putative annotations supported by both database and spectral-library searching are summarized in Table 6 and include sucrose, maltotriose, myo-inositol, citric acid, pyroglutamic acid, and choline. Sucrose was annotated in both positive- and negative-ionization modes. For transparency, the complete retained compound-identification output, including database-only assignments, is provided in Table S2.

The representative polar compounds presented in Table 6 eluted early during reversed-phase chromatographic separation, with retention times ranging from 1.189 to 1.790 min. However, the complete compound-identification output extended to retention times of 42.929 min in positive-ionization mode and 37.887 min in negative-ionization mode (Table S2). Thus, the early retention times of the compounds presented in Table 6 reflect the selection of highly polar representative compounds rather than the chromatographic coverage of the overall analysis.

Free glucose and fructose were not among the compound-identification hits in the retained MassHunter reports. Their absence from the annotation output should not be interpreted as evidence of their absence from cocoa pulp juice. Because corresponding fermented samples were not analyzed by LC–QTOF-MS, the present data cannot establish substrate consumption, fermentation-induced metabolite production, or associations between individual metabolites and the observed antimicrobial activity.

## 3. Discussion

The findings of the present study demonstrate that cocoa pulp juice can be repurposed from an underutilized cocoa-processing by-product into a promising fermentation substrate for *L. paracasei* WU2502. This observation is particularly relevant because previous studies involving cocoa-derived by-products have largely concentrated on physicochemical characterization, waste management, or their use as alternative food ingredients [2]. The ability of *L. paracasei* WU2502 to grow efficiently in cocoa pulp juice suggests a strong compatibility between the nutritional profile of the substrate and the metabolic capacity of the strain. Rather than functioning simply as a passive growth medium, cocoa pulp juice appears to provide a chemically favorable environment containing fermentable sugars, organic acids, and bioactive metabolites that may collectively support probiotic metabolism and functional activity [1,6].

The LC–QTOF-MS analysis provided baseline chemical characterization of the unfermented cocoa pulp juice used as the starting fermentation substrate. Representative putative annotations included sucrose, maltotriose, myo-inositol, citric acid, pyroglutamic acid, and choline. These findings characterize selected constituents of the starting matrix but do not establish their utilization during fermentation. These findings are consistent with previous studies describing cocoa pulp juice or cocoa honey as a sugar-rich by-product with potential industrial and functional food applications [1,19]. In fruit-based LAB fermentations, fermentable carbohydrates serve not only as energy sources but also as precursors for organic acid production and secondary metabolite formation [20]. However, because corresponding fermented samples were not analyzed by LC–QTOF-MS in the present study, changes in individual metabolites, substrate consumption, and fermentation-induced metabolite production could not be determined. Thus, the LC–QTOF-MS data should be interpreted as baseline characterization of the starting matrix rather than as evidence that specific annotated compounds were utilized by WU2502 during fermentation. Glucose supplementation was included to evaluate whether an additional readily fermentable carbon source could influence the growth of WU2502 in cocoa pulp juice. In previous studies, exogenous glucose supplementation has been used to support or evaluate LAB growth in plant-derived fermentation matrices [21]. However, the effect of glucose supplementation may depend on the fermentation matrix and experimental conditions. In the present study, supplementation with 1% glucose did not consistently increase the final viable counts of WU2502 compared with the corresponding unsupplemented formulations, indicating that additional glucose did not consistently confer a growth advantage under the conditions tested.

The predicted carbohydrate-utilization characteristics of WU2502 are compatible with, but do not directly explain, the observed growth and acidification in cocoa pulp juice. However, because gene expression and enzyme activity were not directly measured, these genomic observations should be interpreted as predicted functional potential rather than evidence of the mechanisms underlying the observed fermentation phenotype. The presence of genes encoding predicted glycoside hydrolases and glycosyltransferases, including members of the GH32, GH13, GH1, GT2, and GT4 families, indicates genomic potential related to carbohydrate utilization and glycan metabolism. GH32 enzymes comprise fructan-active hydrolases and transferases involved in the metabolism of sucrose-derived fructans and fructooligosaccharides [22,23]. GT2 and GT4 glycosyltransferases are often involved in exopolysaccharide synthesis and may contribute to stress adaptation, cell surface modification and survival under environmental stress related to fermentation [24]. These genetic features indicate predicted functional capacities that may facilitate adaptation of WU2502 to a plant-derived, carbohydrate-rich fermentation matrix; however, their specific contribution to growth in cocoa pulp juice was not experimentally demonstrated. Although many of the genomic features identified in WU2502 are commonly found among *L. paracasei* strains, previous comparative genomic studies have shown that strains within this species share a conserved core genome but differ in accessory genes related to carbohydrate utilization, stress adaptation, exopolysaccharide biosynthesis, CRISPR systems, and antimicrobial functions, reflecting adaptation to different ecological niches [18,25]. Therefore, rather than identifying unique genomic determinants, the present study characterizes the predicted genomic potential of WU2502 alongside its observed fermentation and antimicrobial phenotypes, without establishing direct functional relationships between these genomic features and the observed activities. Future comparative genomic analyses with additional *L. paracasei* strains will further clarify strain-specific genetic traits associated with substrate adaptation and functional properties.

In addition to carbohydrate metabolism, tolerance to acidic and phytochemical-related stress is important for microbial persistence in cocoa pulp juice. Cocoa pulp juice is naturally acidic and contains several organic acids, including citric acid, which may impose physiological stress on fermenting microorganisms [1]. Genome annotation identified multiple genes associated with acid tolerance and environmental stress responses, including F_0_F_1_-ATP synthase subunits, molecular chaperones, and oxidative stress-related proteins. The F_0_F_1_ ATPase system is a major acid resistance mechanism in lactic acid bacteria, as it contributes to internal pH homeostasis through proton extrusion under acidic conditions [26]. Likewise, molecular chaperones (*DnaK*, *DnaJ*, *ClpB*, *ClpP*) may contribute to protein folding, refolding, and protection against stress-induced protein damage [27]. Genes involved in oxidative-stress responses, such as *sodA* and *tpx*, may contribute to the ability of WU2502 to cope with oxidative conditions encountered during growth and fermentation. Similar defense mechanisms, including thioredoxin peroxidase (*tpx*), have been reported in *L. paracasei* strains [28]. Overall, these genetic features indicate predicted stress-response capacities that are consistent with stress-associated traits previously reported in genomic studies of potential probiotic *L. paracasei* strains; however, their functional contributions in WU2502 require experimental validation [29].

The antimicrobial activity observed against foodborne pathogens may result from multiple synergistic mechanisms associated with probiotic fermentation. Organic acids produced by lactic acid bacteria can reduce environmental pH, diffuse across bacterial membranes in an undissociated form, disrupt intracellular pH homeostasis, and interfere with essential metabolic pathways. Nevertheless, antimicrobial activity in fermented systems is rarely attributable to acidification alone [30]. In the present study, genome mining analysis identified putative bacteriocin-associated biosynthetic genes encoding Carnocin CP52 and Thermophilin A. In addition, antiSMASH analysis predicted a RiPP-like biosynthetic region showing similarity to previously reported antimicrobial peptides, including gassericin T, gassericin E, coagulin, carnobacteriocin XY, salivaricin CRL1328, and lactococcin G. BAGEL4 and antiSMASH are widely used bioinformatic platforms for identifying bacteriocin- and RiPP-associated biosynthetic gene clusters in bacterial genomes, and these systems are increasingly recognized as important contributors to antimicrobial activity and food biopreservation [31,32]. These findings indicate the genetic potential of WU2502 for bacteriocin biosynthesis; however, the predicted bacteriocin-associated genes were not experimentally confirmed at the peptide level.

Preliminary neutralization and Proteinase K sensitivity assays suggested a possible pH-dependent contribution to the antimicrobial activity, as neutralization markedly reduced the inhibitory effect, whereas Proteinase K treatment had little effect. However, because these assays were qualitative, the relative contributions of acidic conditions, proteinaceous compounds, and other antimicrobial factors could not be determined. Therefore, the bacteriocin-associated genes and RiPP-like biosynthetic regions identified by genome mining should be regarded as putative biosynthetic potential rather than evidence of active bacteriocin production. Further peptide purification and characterization are required to validate these predicted antimicrobial compounds. It has been reported that the fermentation of Cocoa pulp juice by *Laetiporus persicinus* produced a new beverage with a tropical aroma [33]. Therefore, cocoa pulp juice is a candidate substrate for potential food applications. In the present study, the MIC and MBC values of the fermented cocoa pulp juice against the pathogens showed moderate concentrations (12.5–25% *v*/*v*). However, these relatively high effective concentrations indicate that further studies in food matrices are required to determine whether the observed antimicrobial activity can be translated into practical food applications without adversely affecting sensory or technological properties.

The absence of detected acquired antimicrobial resistance genes and major virulence-associated determinants provides supportive genomic evidence for the potential use of WU2502 in food fermentation. This is particularly relevant for starter cultures intended for food applications, where the absence of acquired antimicrobial resistance determinants is an important safety consideration [34,35]. However, genomic screening alone is insufficient to establish safety for industrial applications, and further phenotypic safety assessments are required before industrial-scale use. Furthermore, CRISPR-associated elements may contribute to defense against foreign genetic elements and bacteriophage infection. Collectively, genomic characterization, CAZyme prediction, fermentation experiments, and antimicrobial assays provide complementary evidence supporting the ability of *L. paracasei* WU2502 to grow and acidify cocoa pulp juice and highlight its potential as a candidate starter culture for plant-derived fermentation. Further studies under food-relevant processing conditions are required to establish its technological performance and safety for industrial applications.

These findings are encouraging; however, several limitations should be acknowledged. LC–MS/MS analysis was performed using an untargeted metabolomics approach, providing qualitative rather than absolute metabolite quantification. Moreover, LC–QTOF-MS analysis was performed only on unfermented cocoa pulp juice; therefore, substrate consumption and fermentation-induced metabolite production could not be determined. In addition, bacteriocin-associated genes and RiPP-like biosynthetic regions identified by genome mining were not experimentally validated. The preliminary neutralization and Proteinase K assays were qualitative; therefore, the relative contributions of acidic conditions, proteinaceous compounds, and other antimicrobial factors could not be determined. Furthermore, the sensory properties, storage stability, and performance of fermented cocoa pulp juice in real food matrices were not evaluated. The viability of WU2502 during refrigerated storage and at the potential time of consumption was also not assessed; therefore, its persistence in the fermented product during storage remains to be determined. The relatively high MIC/MBC values also suggest that substantial amounts of fermented cocoa pulp juice may be required for practical food applications, potentially affecting sensory quality and consumer acceptance. Finally, this study did not include a direct comparison with established commercial probiotic strains; therefore, the findings should be regarded as a proof-of-concept demonstrating the feasibility of fermenting cocoa pulp juice using the potential probiotic strain *L. paracasei* WU2502 rather than evidence of superiority over commercial cultures. Future studies should address these limitations through paired analysis of unfermented and fermented samples, targeted metabolomics, functional validation, refrigerated-storage viability assessment, food application studies, and comparative evaluations with commercial starter cultures.

## 4. Materials and Methods

### 4.1. Plant Material Preparation

Fresh cocoa fruits were obtained from a cocoa plantation in Tha Sala District, Nakhon Si Thammarat, Thailand. The fruits were washed thoroughly with sterile distilled water, and the cocoa pulp juice was separated aseptically from the fruit. The obtained cocoa pulp juice was filtered through Whatman No. 1 filter paper to remove suspended particles and debris and was subsequently autoclaved at 121 °C for 15 min. After cooling to room temperature, the sterilized cocoa pulp juice was prepared at concentrations of 25%, 50%, and 100% (*v*/*v*) using sterile distilled water. In addition, a parallel set of formulations was supplemented with 1% glucose to evaluate the effect of an additional readily fermentable carbon source on bacterial growth during fermentation. All prepared samples were stored at 4 °C or used immediately for further experiments [36].

### 4.2. Bacterial Culture and Growth Conditions

*L. paracasei* WU2502 was used in this study. The bacterium was isolated from Palmyra palm sugar and characterized as a potential probiotic strain by our research team [10]. The strain was cultured on de Man, Rogosa and Sharpe (MRS) agar (HiMedia, Mumbai, India), followed by incubation in MRS broth (HiMedia, India) as previously described [37]. The cultures were incubated under static conditions in ambient air at 37 °C for 24–48 h before use in subsequent experiments. Pathogenic bacteria used in this study included *Staphylococcus aureus* ATCC 25923, *Escherichia coli* O157:H7, *Bacillus cereus* WU22001, and *Salmonella enterica* serovar Typhimurium DMST 15674. *B. cereus* WU22001 and *S*. Typhimurium DMST 15674 were kindly provided by Assoc. Prof. Veeranoot Nissapatorn, Walailak University, Thailand. *E. coli* O157:H7 strain E5.4 was received from Assistant Professor Dr. Sakaoporn Prachantasena, Prince of Songkla University. *S. aureus* was purchased from the American Type Culture Collection. All pathogens were cultured on Tryptic Soy Agar (TSA; Difco Laboratories, Detroit, MI, USA) and Tryptic Soy Broth (TSB; Difco Laboratories, Detroit, MI, USA) at 37 °C for 24 h. All bacterial strains were maintained in TSB supplemented with 20% glycerol and stored at −80 °C until use.

### 4.3. Effects of Cocoa Pulp Juice on the Growth of L. paracasei WU2502

The growth of *L. paracasei* WU2502 in cocoa pulp juice formulations was evaluated during fermentation. Briefly, Cocoa pulp juice was prepared at concentrations of 25%, 50%, and 100% (*v*/*v*), with and without supplementation with 1% glucose. Cells of *L. paracasei* WU2502 were grown in MRS broth, harvested by centrifugation, washed with sterile phosphate-buffered saline (PBS), and resuspended before inoculation into the cocoa pulp juice formulations. The inoculum was adjusted to obtain an initial bacterial concentration of approximately 10^5^ CFU/mL. Fermentation was performed using 200 mL of each cocoa pulp juice formulation per flask under static conditions in ambient air at 37 °C for 6 days. Three independent fermentation flasks were prepared for each treatment. Samples for viable cell counts and pH measurements were collected repeatedly from each fermentation flask on days 0, 2, 4, and 6 [36,38].

### 4.4. Antimicrobial Activity of Fermented Cocoa Pulp Juice Using L. paracasei WU2502 Against Pathogens

Cell-free supernatants (CFS) collected on day 6, the final day of fermentation, were used for antimicrobial activity assays. Fermented samples were centrifuged to remove bacterial cells, and the resulting supernatants were filtered through a 0.22 µm membrane filter before use in the agar well diffusion and MIC/MBC assays.

#### 4.4.1. Agar Well Diffusion Assay

The antimicrobial activity of fermented cocoa pulp juice was evaluated using the agar well diffusion method [39]. Briefly, pathogenic bacterial suspensions adjusted to approximately 10^6^ CFU/mL were spread uniformly onto Mueller–Hinton agar (MHA) plates. Wells with a diameter of 6 mm were made using a sterile cork borer, and 100 µL of fermented cocoa pulp juice samples were added into each well. The plates were incubated at 37 °C for 24 h, after which the inhibition zone diameters were measured in millimeters (mm). All experiments were performed in triplicate.

#### 4.4.2. Determination of Minimum Inhibitory Concentration (MIC) and Minimum Bactericidal Concentration (MBC) of Fermented Cocoa Pulp Juice Against Pathogens

The MIC and MBC values of fermented cocoa pulp juice against pathogenic bacteria were determined using the broth microdilution method [40]. Two-fold serial dilutions of fermented cocoa pulp juice samples were prepared in Mueller-Hinton Broth (MHB) to obtain different concentrations. The concentrations reported for the MIC and MBC assays (%, *v*/*v*) represent the final concentrations of CFS in each well after mixing with the bacterial suspension. The final bacterial inoculum in each well was approximately 5 × 10^5^ CFU/mL. The microplates were incubated at 37 °C for 24 h. The MIC was defined as the lowest concentration showing no visible bacterial growth. For MBC determination, 100 µL from wells showing no visible growth were plated onto Mueller–Hinton agar and incubated at 37 °C for 24 h. The MBC was defined as the lowest concentration at which no bacterial growth was observed after subculture. MIC and MBC determinations were performed in three independent experiments.

#### 4.4.3. Preliminary Assessment of Antimicrobial Mechanisms

The antimicrobial substances produced during fermentation were preliminarily evaluated according to [41] with slight modifications. Briefly, supernatants of fermented cocoa pulp juice were collected and divided into three conditions: untreated, pH-neutralized, and Proteinase K-treated. For pH neutralization, the supernatant was adjusted to pH 7.2 using sterile 1 N NaOH. For proteolytic enzyme treatment, the supernatant was treated with Proteinase K at a final concentration of 1 mg/mL and incubated at 30 °C for 120 min, followed by heating at 80 °C for 10 min to inactivate the enzyme. All untreated and treated supernatants were filtered through 0.2 µm membrane filters. Antimicrobial activity was evaluated using the agar well diffusion assay on MHA. Following incubation at 37 °C for 24 h, inhibition zones were measured in millimeters (mm).

### 4.5. DNA Extraction and Genomic Analysis

The genomic DNA of *L. paracasei* WU2502 was isolated using the DNeasy Blood & Tissue Kit (QIAGEN, Hilden, Germany) according to the manufacturer’s instructions. DNA quantity and purity were evaluated spectrophotometrically based on the A260/A280 absorbance ratio and further verified by agarose gel electrophoresis.

WGS was performed at the Beijing Genomics Institute (BGI, Beijing, China) using the MGISEQ-2000 platform with 150 bp paired-end sequencing chemistry. Raw sequencing reads were processed through the BacSeq v1.0 pipeline [42], an integrated bioinformatics workflow designed for bacterial genome assembly, annotation, and quality assessment. Genome assembly was conducted using SPAdes v4.3.0 [43], and assembly quality metrics and genome completeness were evaluated using QUAST v 5.3.0 [44] and BUSCO v6.1.0 [45], respectively. The assembled genome was subsequently submitted to the PATRIC Comprehensive Genome Analysis Service and annotated using the RAST toolkit (RASTtk) v1.3.0 [46]. The RASTtk/PATRIC annotation was used for the genome annotation statistics reported in this study, including the numbers of protein-coding sequences (CDSs), tRNAs, and rRNAs.

Putative mobile genetic elements, prophage regions, virulence-associated determinants, and antimicrobial resistance genes (ARGs) were identified using mobileOG-db v1.1.3 [47], PHASTEST v2.0.14 [48], VirulenceFinder v2.0.5 [49], and ResFinder v4.7.2 [50], respectively. ARG identification was conducted using a minimum threshold of ≥90% sequence identity and ≥60% alignment coverage. CRISPR-Cas loci were predicted using CRISPRCasFinder [51]. Additionally, genes encoding ribosomally synthesized and post-translationally modified peptides, including bacteriocin-associated biosynthetic clusters, were predicted using the BAGEL4 web server [52]. Secondary metabolite biosynthetic gene clusters were further analyzed using antiSMASH 8.0 [53].

Genomic relatedness was further assessed through average nucleotide identity (ANI) analysis using FastANI, with the reference genome selected automatically by the analysis pipeline.

### 4.6. Carbohydrate-Active Enzymes Analysis

CAZymes in *L. paracasei* WU2502 were predicted using the dbCAN server based on the annotated protein sequences [54]. The protein FASTA file generated from genome annotation was submitted to dbCAN for CAZyme classification. Based on the available annotation output, 37 genes were assigned to eight explicitly quantified CAZyme families: GT2, GT4, GH25, GH13_31, GH1, GH32, GH36, and CE9. The predicted CAZyme families were manually curated and interpreted based on their putative roles in carbohydrate utilization, polysaccharide metabolism, and cell wall remodeling.

### 4.7. LC-MS/MS Analysis

LC–MS/MS analysis was conducted to provide baseline chemical characterization of the unfermented cocoa pulp juice used as the starting fermentation substrate [55]. Cocoa pulp juice samples were prepared by dissolving the samples in LC–MS-grade water to a final concentration of approximately 10 mg/mL, followed by filtration through a 0.2 µm nylon syringe filter before analysis. Untargeted metabolite profiling was performed using an Agilent 1290 Infinity II Liquid Chromatography system coupled to an Agilent 6545 Quadrupole Time-of-Flight Mass Spectrometer (LC-QTOF-MS; Agilent Technologies, Santa Clara, CA, USA) equipped with a dual Agilent Jet Stream electrospray ionization (Dual AJS-ESI) source operating in both positive and negative ionization modes (Auto MS/MS). Chromatographic separation was carried out on a ZORBAX Eclipse Plus C18 Rapid Resolution HD column (100 × 2.1 mm, 1.8 µm; Agilent Technologies) maintained at 25 °C. The mobile phases consisted of 0.1% formic acid in water (A) and 0.1% formic acid in acetonitrile (B) at a flow rate of 0.20 mL/min. The gradient program was as follows: 95% A/5% B from 0 to 5 min, followed by a linear gradient to 0% A/100% B at 35 min, which was maintained until 40 min; the gradient was then returned to 95% A/5% B at 45 min and maintained until 50 min for re-equilibration. The injection volume was 2 µL. Mass spectra were acquired over an m/z range of 100–1500 for MS and 50–1500 for MS/MS using collision energies of 10, 20, and 40 eV. Instrument parameters included a gas temperature of 325 °C, gas flow of 13 L/min, nebulizer pressure of 35 psig, sheath gas temperature of 275 °C, sheath gas flow of 12 L/min, capillary voltage of 4000 V, nozzle voltage of 2000 V, fragmentor voltage of 175 V, skimmer voltage of 65 V, and octopole RF peak of 750 V.

Raw data were processed using MassHunter Workstation Qualitative Analysis Navigator (Version 8.0), and metabolite annotation was performed by comparison with the METLIN Personal Compound Database and Library (PCDL, Version 8).

### 4.8. Statistical Analysis

All experiments were performed in triplicate, and data are presented as mean ± standard deviation (SD). Statistical analyses were carried out using three-way mixed ANOVA with incubation time as the repeated factor and Greenhouse–Geisser correction, followed by Sidak-adjusted pairwise comparisons. Normality, homogeneity of variance and sphericity were checked using the Shapiro–Wilk, Levene’s and Mauchly’s tests, respectively. Differences were considered significant at *p* < 0.05 (IBM SPSS Statistics, version 27).

## 5. Conclusions

This study demonstrated that cocoa pulp juice can serve as a promising natural substrate for fermentation by the potential probiotic *L. paracasei* WU2502. The strain exhibited strong growth, antibacterial activity against foodborne pathogens, and genomic features associated with carbohydrate utilization, environmental stress adaptation, and antimicrobial potential. Genome analysis also supported its preliminary safety profile, as no acquired antimicrobial resistance genes or major virulence-associated determinants were detected. In addition, LC–MS/MS profiling confirmed that cocoa pulp juice contains diverse fermentable sugars and bioactive metabolites that may support microbial growth and fermentation. Overall, these findings highlight the potential of cocoa pulp juice valorization for developing sustainable fermented food products and natural biopreservation strategies. Future studies should validate the antimicrobial mechanisms through targeted metabolomic and proteomic analyses, evaluate product stability and sensory quality, and investigate the performance of the fermented product in real food systems to facilitate industrial application.

## Figures and Tables

**Table 1:** Viability of *Lacticaseibacillus paracasei* WU2502 during fermentation in cocoa pulp juice formulations.

Day	Log CFU/mL					
	Cocoa pulp juice alone (%)			Cocoa pulp juice (%) plus 1% glucose		
	25%	50%	100%	25%	50%	100%
Day 0	5.53 ± 0.03 ^aA^	5.49 ± 0.05 ^aA^	5.44 ± 0.02 ^aA^	5.51 ± 0.02 ^aA^	5.41 ± 0.10 ^aA^	5.10 ± 0.09 ^aB^*
Day 2	6.51 ± 0.24 ^bB^	7.84 ± 0.21 ^bA^	8.36 ± 0.17 ^bA^	7.42 ± 0.10 ^bC^*	8.00 ± 0.04 ^bB^	8.33 ± 0.14 ^bA^
Day 4	10.70 ± 0.04 ^cC^	11.05 ± 0.07 ^cB^	11.29 ± 0.02 ^cA^	10.67 ± 0.06 ^cA^	10.38 ± 0.10 ^cB^*	10.81 ± 0.03 ^cA^*
Day 6	9.54 ± 0.02 ^dC^	11.19 ± 0.05 ^cD^	11.46 ± 0.00 ^cA^	10.59 ± 0.07 ^cB^*	9.05 ± 0.09 ^dC^*	11.18 ± 0.06 ^dA^*

Values are mean ± SD (n = 3, sampled repeatedly). Different lowercase letters within a column indicate significant differences among days; different uppercase letters within a row indicate significant differences among juice concentrations within the same glucose treatment. An asterisk (*) indicates a significant effect of glucose at the same concentration and day (three-way mixed ANOVA, Sidak post hoc, *p* < 0.05).

**Table 2:** Measurement of pH in different formulations of fermented cocoa pulp juice by *Lacticaseibacillus paracasei* WU2502.

Day	pH					
	Cocoa pulp juice alone (%)			Cocoa pulp juice (%) plus 1% glucose		
	25%	50%	100%	25%	50%	100%
Day 0	4.03 ± 0.15 ^aA^	3.92 ± 0.02 ^aA^	3.90 ± 0.10 ^aA^	3.99 ± 0.02 ^aA^	3.95 ± 0.05 ^aA^	3.88 ± 0.02 ^aA^
Day 2	3.35 ± 0.05 ^bA^	3.38 ± 0.03 ^bA^	3.47 ± 0.11 ^bA^	3.31 ± 0.01 ^bB^	3.35 ± 0.00 ^bAB^	3.46 ± 0.11 ^bA^
Day 4	3.12 ± 0.08 ^cA^	3.15 ± 0.05 ^cA^	3.21 ± 0.09 ^cA^	3.08 ± 0.01 ^cA^	3.12 ± 0.02 ^cA^	3.16 ± 0.01 ^cA^
Day 6	3.15 ± 0.05 ^cA^	3.17 ± 0.03 ^cA^	3.23 ± 0.07 ^cA^	3.11 ± 0.04 ^cA^	3.14 ± 0.02 ^cA^	3.17 ± 0.02 ^cA^

Values are mean ± SD (n = 3, sampled repeatedly). Different lowercase letters within a column indicate significant differences among days; different uppercase letters within a row indicate significant differences among juice concentrations within the same glucose treatment (three-way mixed ANOVA, Sidak post hoc, *p* < 0.05).

**Table 3:** Antimicrobial activity of fermented cocoa pulp juice using *L. paracasei* WU2502 against pathogens using agar well diffusion assay and broth microdilution assay.

Pathogens	Agar Well Diffusion Assay (Zone Diameter; mm)			
	Cocoa Pulp Juice (%)		Cocoa Pulp Juice (%) with 1% Glucose	
	100%	50%	100%	50%
*B. cereus*	14.67 ± 0.58	13.00 ± 1.00	14.00 ± 1.00	12.00 ± 1.00
*S. aureus*	12.33 ± 1.15	10.33 ± 0.58	10.00 ± 1.00	11.00 ± 1.00
*S.* Typhimurium	14.67 ± 0.58	11.33 ± 0.58	14.67 ± 0.58	13.33 ± 0.58
*E. coli* O157:H7	13.33 ± 0.58	12.00 ± 1.00	13.67 ± 0.58	12.67 ± 0.58
	MIC/MBC values (% *v*/*v*)			
	**Cocoa pulp juice (%)**		**Cocoa pulp juice (%) with 1% glucose**	
	100%	50%	100%	50%
*B. cereus*	12.5/12.5	12.5/12.5	12.5/12.5	12.5/12.5
*S. aureus*	12.5/25	12.5/25	12.5/25	12.5/25
*S.* Typhimurium	12.5/12.5	12.5/12.5	12.5/12.5	12.5/12.5
*E. coli* O157:H7	12.5/25	12.5/25	12.5/25	12.5/25

**Table 4:** Genome features of *L. paracasei* WU2502.

Genome Feature	WU2502
Bases (bp)	3,019,699
Number of contigs	50
L50	10
N50	108,079
GC content (%)	46.27
Number of Coding Sequences (CDS)	3114
CRISPR	1
rRNA	5
tRNA	50
tmRNA	1
Classification	*Lacticaseibacillus paracasei* subsp. *paracasei* GCF_000829035.1 (ASM82903v1)
FastANI reference genome accession	GCF_000829035.1
Average nucleotide identity (ANI)	98.24

**Table 5:** Selected genes associated with stress adaptation, carbohydrate metabolism, cell-surface traits, and antimicrobial biosynthetic potential in *L. paracasei* WU2502.

Functional Category	Gene/Locus Tag	Possible Probiotic Relevance
Acid tolerance	*atpA*, *atpB*, *atpD*, *atpE*, *atpF*, *atpG*/AMNHOHEI_01959–01965	Acid stress adaptation and intracellular pH homeostasis
Stress response	*dnaK*, *dnaJ*, *clpB*, *clpP*/AMNHOHEI_00611, AMNHOHEI_00612, AMNHOHEI_00860, AMNHOHEI_02318	Protein folding and survival under environmental stress
Oxidative stress protection	*sodA*, *tpx*, *npr*,/AMNHOHEI_00967, AMNHOHEI_00805, AMNHOHEI_01099, AMNHOHEI_00055	Protection against oxidative stress
Osmotic/GI stress adaptation	*mscL*, *nhaK*, *gbuA*, *gbuB*, *gbuC*/AMNHOHEI_00007, AMNHOHEI_01501, AMNHOHEI_01326–01328	Osmotic balance and stress survival
Cell surface/EPS-related functions	*epsJ*, *epsH*/AMNHOHEI_01321, AMNHOHEI_02490, AMNHOHEI_01066, AMNHOHEI_02116	Cell surface modification, adhesion, and biofilm-related traits
Carbohydrate utilization	*lacD*, *scrB*, *manP*, *gatB*, *gatC*, *malX*/AMNHOHEI_00025, AMNHOHEI_02018, AMNHOHEI_00089, AMNHOHEI_00087–00088, AMNHOHEI_00134	Utilization of plant-derived sugars during fermentation
Antimicrobial potential	Carnocin CP52, Thermophilin A; AMNHOHEI_01204	Potential contribution to antimicrobial activity

**Table 6:** Representative putatively annotated compounds in unfermented cocoa pulp juice by LC–QTOF-MS.

Chemical Class	Putative Annotation	Mode	RT (min)	Precursor m/z	Mass Error (ppm)	MS/MS Base Peak m/z	Formula	DB Score	Library Score	Relative Peak Height
Carbohydrate (disaccharide)	Sucrose	Negative	1.395	341.1088	0.40	59.0135	C12H22O11	99.43	98.27	22,340,077
Carbohydrate (disaccharide)	Sucrose	Positive	1.401	365.1061	1.83	365.1058	C12H22O11	98.25	99.82	26,000,775
Carbohydrate (oligosaccharide)	Maltotriose	Positive	1.439	527.1590	1.41	527.1577	C18H32O16	97.71	83.27	273,798
Sugar alcohol (cyclitol)	myo-Inositol	Positive	1.314	203.0529	1.43	203.0527	C6H12O6	98.64	88.97	479,581
Organic acid	Citric acid	Negative	1.784	191.0190	3.80	111.0081	C6H8O7	97.22	95.24	1,701,361
Amino-acid derivative	Pyroglutamic acid	Positive	1.790	130.0502	2.54	84.0444	C5H7NO3	86.95	88.09	381,030
Quaternary ammonium compound	Choline	Positive	1.189	104.1072	—	104.1071	C5H14NO	87.34	94.93	1,275,566

Footnote: Compounds shown are representative putative annotations supported by database and spectral-library searching and selected for baseline characterization of the cocoa pulp juice matrix. Relative peak height is presented as a semi-quantitative analytical signal and does not represent absolute metabolite concentration. Compound assignments should be interpreted as putative annotations rather than definitive structural identifications.

## Data Availability

The original contributions presented in this study are included in the article/Supplementary Material. Further inquiries can be directed to the corresponding author. The whole-genome shotgun sequence of *Lacticaseibacillus paracasei* WU2502 has been deposited in GenBank under accession number JBYUPA000000000.1. The LC–MS/MS raw data have not been deposited in a public repository and are available from the corresponding author upon reasonable request.

## Supplementary Materials

The following supporting information can be downloaded at: https://www.mdpi.com/article/10.3390/ijms27219795/s1.
