# Supplementary material for: Cocoa Pulp Juice as a Fermentation Substrate for Potential Probiotic Lacticaseibacillus paracasei WU2502: Genomic Characterization, Metabolite Profiling, and Antimicrobial Activity Against Foodborne Pathogens
Source: Int J Mol Sci. 2026 Oct 31;27(21):9795. doi: 10.3390/ijms27219795 (PMC13762919; doi:10.3390/ijms27219795)
Supplement: Supplementary file 1 [file ijms-27-09795-s001.zip › ijms-4624108-supplementary.pdf]

Table S1. Predicted carbohydrate-active enzyme (CAZyme) families identified in *Lacticaseibacillus paracasei* WU2502

**CAZyme family**	**Number of genes**	**Predicted function**
GT2	9	Glycosyltransferase associated with exopolysaccharide biosynthesis and carbohydrate metabolism
GT4	6	Glycosyltransferase involved in sugar transfer and carbohydrate utilization
GH25	6	Lysozyme-like glycoside hydrolase associated with cell wall remodeling
GH13_31	5	α-Glucosidase-related enzyme involved in carbohydrate degradation
GH1	5	β-Glucosidase associated with glycoside hydrolysis
GH32	2	Fructosidase/invertase-related enzyme involved in sucrose and fructose metabolism
GH36	2	α-Galactosidase associated with oligosaccharide metabolism
CE9	2	Carbohydrate esterase involved in carbohydrate modification
PL families	Detected	Polysaccharide degradation and modification
CBM families	Detected	Carbohydrate-binding modules involved in substrate recognition

Abbreviations: GH, glycoside hydrolase; GT, glycosyltransferase; CE, carbohydrate esterase; PL, polysaccharide lyase; CBM, carbohydrate-binding module.

Table S2. Complete compound-identification output from LC–QTOF-MS analysis of unfermented cocoa pulp juice

Mode	Putative annotation	Formula	Precursor m/z	RT (min)	Overall score	DB score
Positive	His-Glu-Lys	C17H28N6O6	413.2131	1.088	89.15	92.62
Positive	Choline	C5H14NO	104.1072	1.189	91.13	87.34
Positive	6-(α-D-Glucosaminyl)-1D-myo-inositol	C12H23NO10	380.0957	1.213	49.19	98.37
Positive	6-(α-D-Glucosaminyl)-1D-myo-inositol	C12H23NO10	364.1214	1.238	49.84	99.68
Positive	myo-Inositol	C6H12O6	203.0529	1.314	93.81	98.64
Positive	Hexandraside E	—	362.0980	1.376	43.85	87.70
Positive	Sucrose	C12H22O11	365.1061	1.401	99.03	98.25
Positive	Maltotriose	C18H32O16	527.1590	1.439	90.49	97.71
Positive	6-Acetyl-D-glucose	—	245.0638	1.589	49.00	97.99
Positive	α,β-Trehalose	C12H22O11	365.1055	1.614	99.77	99.54
Positive	Sarmentosin epoxide	—	314.0851	1.714	48.64	97.29
Positive	Pyroglutamic acid	C5H7NO3	130.0502	1.790	87.52	86.95
Positive	Octulose-1,8-bisphosphate	—	423.0083	1.840	43.41	86.82
Positive	Acetyl-maltose	—	423.0889	1.940	46.93	93.86
Positive	Acetyl-maltose	—	407.1165	1.990	49.32	98.64
Positive	6′-Apiosyllotaustralin	—	416.1526	9.006	49.79	99.58

Mode	Putative annotation	Formula	Precursor m/z	RT (min)	Overall score	DB score
Positive	PI(O-16:0/16:0)	—	814.5775	13.841	44.21	88.43
Positive	Trithionic acid	—	194.9089	18.827	36.06	72.12
Positive	C16 Sphinganine	—	274.2740	19.453	49.98	99.96
Positive	Ethyl 2-benzylacetoacetate	—	221.1173	21.583	47.10	94.20
Positive	2,3-Dihydro-3-hydroxy-6-methoxy-2,2-dimethyl-4H-1-benzopyran-4-one	—	245.0784	21.934	49.19	98.38
Positive	Dipropyl hexanedioate	—	253.1412	24.539	49.65	99.31
Positive	Diethyl decanedioate	—	281.1724	28.736	49.70	99.41
Positive	Diisobutyl phthalate	—	301.1412	29.250	49.29	98.58
Positive	14,14,14-Trifluoro-11Z-tetradecenyl acetate	—	309.2033	31.003	43.23	86.45
Positive	Nordihydrocapsaicin	—	294.2060	32.607	49.57	99.13
Positive	Didodecyl thiobispropanoate	—	537.3943	33.985	48.37	96.74
Positive	TG(14:0/18:0/18:3)[iso6]	—	851.7093	35.163	47.68	95.35
Positive	TG(16:1/17:2/19:1)[iso6]	—	877.7254	35.588	49.04	98.09
Positive	N-Stearoyl valine	—	406.3287	35.814	49.54	99.07
Positive	6β-Hydroxy-3-oxo-5β-cholan-24-oic acid	—	413.2659	37.718	49.61	99.22
Positive	13E-Docosenamide	—	338.3416	39.973	97.42	96.29

Mode	Putative annotation	Formula	Precursor m/z	RT (min)	Overall score	DB score
Positive	TG(14:0/18:0/18:3)[iso6]	—	851.7099	42.090	48.47	96.94
Positive	2,4,6-Triaminotoluene	—	155.1292	42.479	43.13	86.26
Positive	Methenamine	—	141.1131	42.929	88.82	85.61
Negative	6-(α-D-Glucosaminyl)-1D-myo-inositol	—	340.1243	1.207	44.54	89.09
Negative	N-Acetyl-8-O-methyl-neuraminic acid	—	322.1137	1.232	48.34	96.67
Negative	D-Lactose	—	377.0869	1.283	88.17	93.02
Negative	Nephritogenoside	—	539.1381	1.332	49.05	98.09
Negative	Sucrose	$C_{12}H_{22}O_{11}$	341.1088	1.395	98.85	99.43
Negative	Citbismine C	—	683.2248	1.408	40.47	80.94
Negative	D-(+)-Turanose	—	387.1142	1.433	49.66	99.32
Negative	Cyflufenamid	—	457.1193	1.533	46.08	92.16
Negative	D-Erythroascorbic acid 1′-α-D-glucoside	—	353.0723	1.570	49.78	99.57
Negative	Sucrose	$C_{12}H_{22}O_{11}$	341.1086	1.608	98.82	99.30
Negative	Sarmentosin epoxide	—	290.0879	1.633	49.57	99.14
Negative	(R)-(+)-2-Pyrrolidone-5-carboxylic acid	—	128.0354	1.708	43.86	87.72
Negative	(1R,6S)-6-Amino-5-oxocyclohex-2-ene-1-carboxylate	—	200.0565	1.733	49.95	99.90

Mode	Putative annotation	Formula	Precursor m/z	RT (min)	Overall score	DB score
Negative	Citric acid	C6H8O7	191.0190	1.784	96.23	97.22
Negative	2,3-Dihydroxy-2,4-cyclopentadien-1-one	—	111.0082	1.809	42.90	85.79
Negative	Acetyl-maltose	—	383.1194	1.934	49.65	99.30
Negative	Acetyl-maltose	—	429.1248	1.971	49.64	99.29
Negative	Phenylalanyl-Aspartate	—	315.0752	5.492	35.40	70.79
Negative	Psilocybin	—	329.0911	10.102	44.34	88.68
Negative	5-Hydroxypropafenone	—	356.1862	31.999	49.42	98.83
Negative	Vitamin-D-related database hit	—	473.3055	34.605	49.09	98.18
Negative	MGDG(18:0/18:2)	—	827.5868	37.411	46.93	93.85
Negative	Piperonyl sulfoxide	—	383.1892	37.887	43.76	87.52

Footnote: Complete compound-identification output retained from the MassHunter workflow for unfermented cocoa pulp juice analyzed in positive- and negative-ionization modes. The reports contained 35 and 23 compound-identification hits, respectively. These values represent identification hits generated by the applied workflow and not the total number of raw LC–MS features detected before annotation. DB-only assignments, particularly those lacking spectral-library support, are reported for transparency but should be interpreted cautiously and are not considered definitive metabolite identifications. Compounds designated as biologically implausible, potentially exogenous, redundant, or possible contaminants were not used for biological interpretation in the main text.
